# An Unusual Encounter of Skin Condition Mimicking Acrokeratosis Paraneoplastica (Bazex Syndrome)

**DOI:** 10.7759/cureus.17849

**Published:** 2021-09-09

**Authors:** Vikas Vaibhav, Raviprakash Meshram, Tushar Kalonia

**Affiliations:** 1 Forensic Medicine and Toxicology, All India Institute of Medical Sciences, Rishikesh, Rishikesh, IND; 2 Forensic Medicine, All India Institute of Medical Sciences, Rishikesh, Rishikesh, IND; 3 Pathology, All India Institute of Medical Sciences, Rishikesh, Rishikesh, IND

**Keywords:** acrokeratosis paraneoplastica, bazex syndrome, paraneoplastic skin disease, forensic histopathology, post mortem histology

## Abstract

Acrokeratosis paraneoplastica is a rare paraneoplastic skin disease in which there are erythematous violaceous, scaly plaques on the hands, feet, and acral locations. There is a relationship between various carcinomas of the aerodigestive tract and skin eruptions. These were the pioneering work done pertaining to a clinical entity that was showing some inter-relationship between a skin condition and carcinoma anywhere inside the body. Bazex syndrome is mostly associated with carcinomas of the upper aero-digestive tract, but other malignancies were also being reported. In this case, the patient was in advancing age with cachexic features along with liver and lung lesions which prompted us to investigate histologically for evidence of malignancy which came out to be negative. In our case, lungs showed features of pulmonary edema with normal histology. When we examined the liver, gross lesions were present but no evidence of malignancy was noted and the liver showed normal parenchyma histologically. Specimens taken from hand and foot showed hyperkeratosis along with bacterial colonies in the overlying epidermis. The spleen showed red pulp with congestion and hemorrhage. Similarly, sections from the kidneys were showing interstitial inflammation and congestion of blood vessels. Specimens from the brain and heart showed unremarkable histology.

## Introduction

Gougerot et al. in 1922 reported a relationship between squamous cell carcinoma of the tongue and papulosquamous skin eruptions for the first time [1]. The relationship between skin lesions similar to psoriasis and squamous cell carcinoma of the piriform fossa was observed by Bazex et al., in 1965, and hence the nomenclature [2]. A case series published in 1967 showed the relationship between internal tumors and skin findings [3]. Bazex syndrome is mostly associated with carcinomas of the upper aero-digestive tract, but other malignancies were also reported [4,5]. In our case, the cutaneous presentation was similar to Bazex syndrome, so we investigated during autopsy and histologically to establish the diagnosis In 1976, a study postulated an association between an internal malignancy and cutaneous condition; wherein it was stated that if malignancy is successfully treated, associated skin condition follows the same path. They investigated and hypothesized that there was no genetic association between the malignancy and the skin condition [6]. Coffey Jr. et al. suggested that different growth factors like epidermal growth factors (EGF) and insulin-like growth factor (IGF) produced by the tumor cells may cross-react with antigen in skin and tumor. EGF and IGF both act as autocrine growth factors for murine and human keratinocytes [7,8]. Another report proposed the contribution of the immune system, especially autoreactive T-cells in the pathogenesis of Bazex syndrome [9].

In a typical Bazex syndrome, acrokeratosis presents as symmetrical psoriasiform lesions on acral regions like hands, feet nose, and ear [9,10]. There is violaceous erythema, yellowish crusts over the skin. The main clue to the diagnosis is the thickening of soles with keratotic papules. Nails are thickened with dystrophic thickening and subungual hyperkeratosis. Sometimes soles resemble arsenic keratosis [9].

Bazex syndrome develops in three stages in which palms and soles are involved extensively in stages 2 and 3 [5-11]. The underlying growing tumor can cause more widespread erythema of the trunk over the period. A similar condition is observed in disorders like bullous pemphigoid or epidermolysis bullosa acquisita. Other dermatological conditions that may confuse a clinician in diagnosing Bazex syndrome are pityriasis rubrapilaris, lupus erythematodes, Reiter’s disease, hyperkeratotic rhagadiform hand, and hereditary palmoplantar keratosis [12].

## Case presentation

A 57-year-old man presented to the emergency department with a history of a road accident. There was no history of vomiting, loss of consciousness, and seizure; he was managed as per advanced trauma life support guidelines.

On examination, blood pressure was 172/92mmHg, pulse rate was 92/min; airway was patent, respiratory rate 24/min, SPO_2_ 99% on room air, and bilateral crepitations were present over chest on auscultation. Glasgow coma scale score was E4V5M6; bilaterally, pupils were normal and reactive. There were no injuries to the chest or pelvis. Hemoglobin was 9.17g/dL, the blood counts were WBC 11.24 cells/cu mm, the platelet was 152.4 cells/cu mm. Liver function test was SGPT/SGOT-45.2U/L/77U/L, ALP/GGT-256U/L/70.9U/L. Renal function showed blood urea-47.4mg/dL, serum creatinine - 0.85mg/dL, serum Na/K/Cl - 132mmol/L/4.07mmol/L/102mmol/L, PT/INR- 1.03 sec. Viral markers were negative.

**Figure 1:**
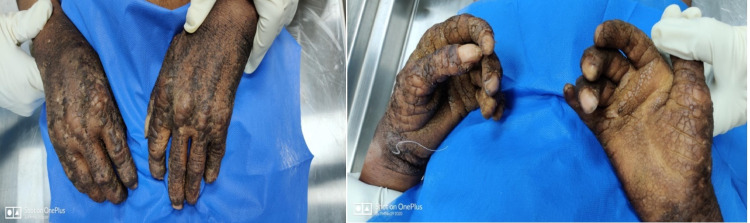
Hands showing symmetrical psoriasiform lesions bilaterally.

**Figure 2:**
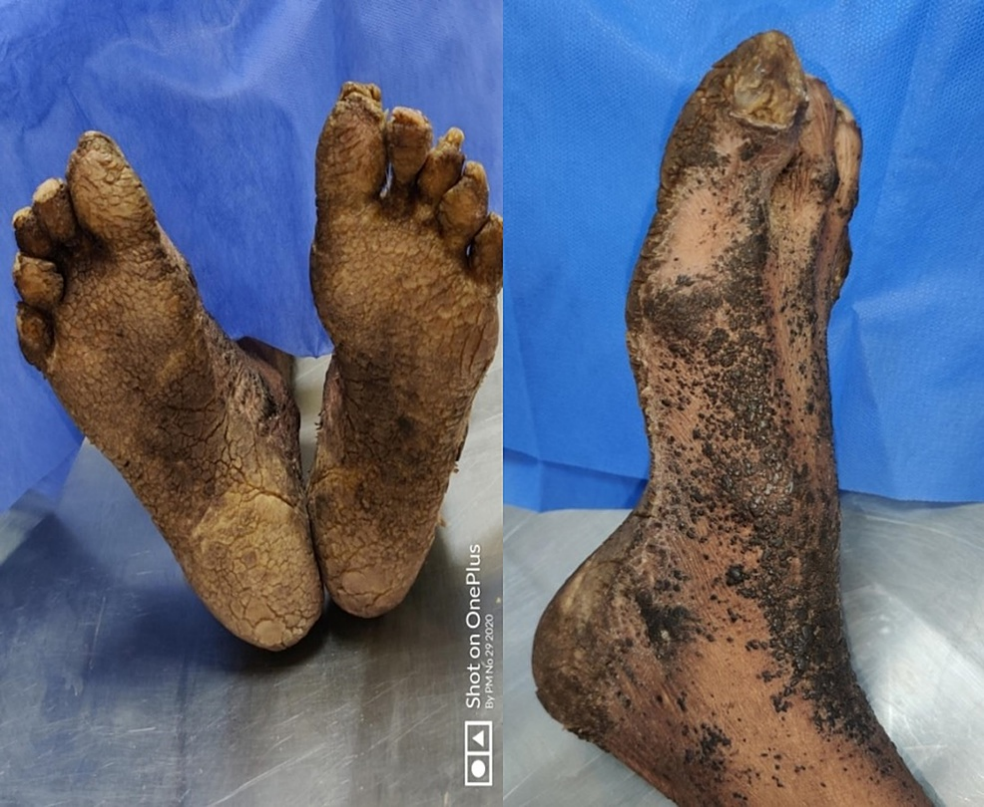
Foot showing symmetrical psoriasiform lesions bilaterally.

Multiple fungating hard, dried and blackened skin lesions were present over both hands and feet along with clubbing in all fingernails lesions were not present over face and ear. Nails were thickened with dystrophic thickening and subungual hyperkeratosis (Figures 1, 2).

During his course of hospital stay of eight days, he developed an acute exacerbation of chronic obstructive pulmonary disease (COPD) for which he was initially put on non-invasive ventilatory support which the patient did not tolerate, then he was intubated and put on mechanical ventilation. He was managed conservatively with intravenous fluids, antibiotics (augmentin, azithromycin, and clindamycin), analgesics, bronchodilators, and other supportive drugs. He also developed right-sided parotitis later. The patient developed respiratory acidosis (pH-7.0) with severe hypercarbia (pco2-64%) and had a cardiac arrest. However, he could not be revived and declared clinically dead, and was subjected to autopsy.

**Figure 3:**
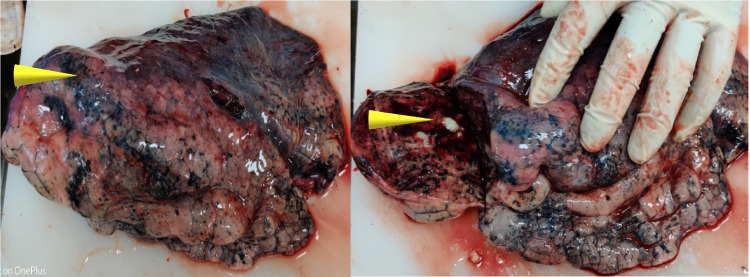
Lungs showing surface with multiple nodules, on cut section thick whitish mucoid material was present marked with arrows.

**Figure 4:**
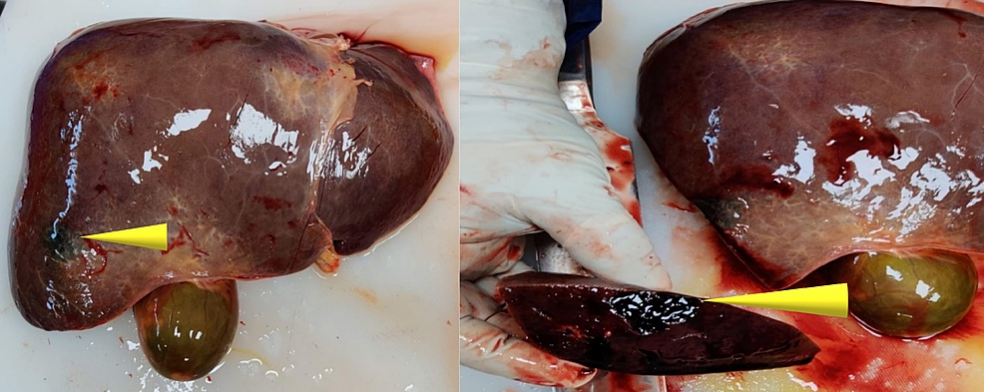
Liver showing dark-colored lesion over the lower aspect of the anterior part of the right lobe of liver marked with arrows.

Due to the peculiar symmetrical distribution of the keratotic lesions and the advancing age of the person, an investigation was focused on associated aero-digestive tract carcinoma or any other malignancy. Weights of lungs were 540g and 530g of left and right sides, respectively. Both the lungs were pale with multiple hard, nodular lesions present over the surface (Figure 3). On cut section blood mixed fluid was observed with multiple pus pockets containing yellowish mucoid cheesy material as depicted by the arrows in Figure 3. The liver was weighing 1,350g and was congested with a dark-colored lesion measuring 4cm x 3cm x 2cm over the lower aspect of the anterior part of the right lobe of the liver as depicted in the anterior view and cut section of the liver in Figure 4. The gall bladder was distended with bile. Kidneys were weighing 120g and 130g right and left, respectively. Kidneys were congested and cortico-medullary differentiation was lost along with a cystic lesion over the lower pole of the left kidney over the medullary region. The spleen was weighing 110g and showed features of congestion. Excisional biopsy specimens from both foot and hands, along with samples from the lesion in the liver, lungs, spleen, and kidney were histologically examined; and no malignancy was detected.

## Discussion

The diagnosis of Bazex syndrome can be perplexing because of very non-specific histopathological findings and clinical features overlapping with clinical entities like psoriasis, Pityriasis rubrapilaris, lupus erythematodes, Reiter’s disease, hyperkeratotic rhagadiform hand, hereditary palmoplantar keratosis [5-13]. However many systemic reviews reported roughly 50%-75% cases having preceding skin manifestation before malignancy. Most of the studies showed hyperkeratosis, acanthosis, parakeratosis, spongiosis, and mixed infiltrate of leukocytes, neutrophils, eosinophils, or mononuclear cells in the dermal layer [13]. In a systemic review done by Rabler et al. in 2017, it was observed that sites involved in Bazex syndrome are ear (28%), nose (33%), fingers (29%), hands (38%), feet (30%), and nails (57%) of the patients [13]. On histopathology, the features of Bazex syndrome most frequently encountered features are hyperkeratosis, parakeratosis, acanthosis, spongiosis, dyskeratotic keratinocytes with vacuolization of the basal layer, and many perivascular inflammatories infiltrates in the dermis, which ranges from leucocytes, eosinophils, neutrophils, or mononuclear cells. Sometimes histological specimens from Bazex syndrome may show interface dermatitis, which may look like lichenoid diseases.

**Figure 5:**
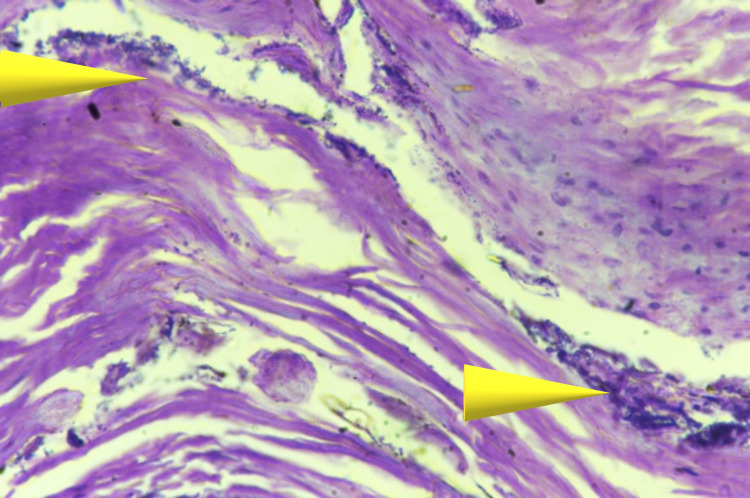
Section taken from foot showing hyperkeratosis with parakeratosis along with numerous bacterial colonies in the epidermis (hematoxylin and eosin stain, magnification - 400x).

**Figure 6:**
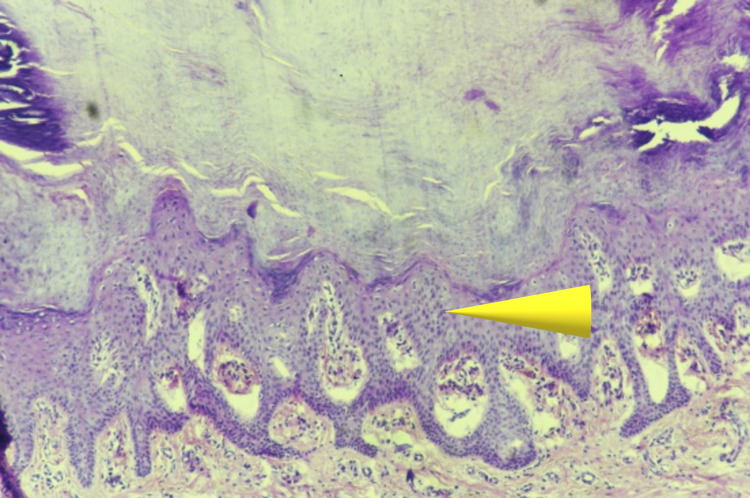
Section taken from hand epidermis having hyperkeratosis with focal parakeratosis and spongiosis with underlying dermis showing mild perivascular lymphocytic infiltrate (hematoxylin and eosin stain, magnification - 400x).

In this case, the patient was in advancing age with cachexic features along with liver and lung lesions which prompted us to investigate histologically for evidence of malignancy which came out to be negative. Bazex syndrome is frequently associated with lung carcinoma as seen in many studies [14-17]. In our case, lungs showed features of pulmonary edema with normal histology. When we examined the liver, gross lesions were present but no evidence of malignancy was noted and the liver showed normal parenchyma histologically. Specimens taken from hand and foot showed hyperkeratosis along with bacterial colonies in the overlying epidermis as depicted by arrows in Figures 5, 6. The spleen showed red pulp with congestion and hemorrhage. Similarly, sections from the kidneys were showing interstitial inflammation and congestion of blood vessels. Specimens from the brain and heart showed unremarkable histology.

## Conclusions

A case of cutaneous manifestation resembling Bazex syndrome, in an advancing age with cachexic features was investigated for the presence of internal malignancy. Even with gross autopsy findings suggestive of some pathology, histological investigations for malignancy were negative. Because of the non-specific histopathological findings and clinical symptoms that can overlap with other clinical entities, diagnosing Bazex syndrome can be difficult. Histopathological analysis of different organs which can be involved and a clinical external examination can help us in diagnosing Bezex syndrome.
